# Cantharidin‐loaded functional mesoporous titanium peroxide nanoparticles for non‐small cell lung cancer targeted chemotherapy combined with high effective photodynamic therapy

**DOI:** 10.1111/1759-7714.13414

**Published:** 2020-04-04

**Authors:** Kun Zheng, Runze Chen, Yanxue Sun, Zhenquan Tan, Ye Liu, Xiao Cheng, Junke Leng, Zhaoming Guo, Pengcheng Xu

**Affiliations:** ^1^ School of Life Science and Medicine Dalian University of Technology Panjin China; ^2^ Department of Pharmaceutical Engineering, College of Pharmacy Inner Mongolia Medical University Hohhot China; ^3^ School of Petroleum and Chemical Engineering Dalian University of Technology Panjin China

**Keywords:** Cantharidin, non‐small cell lung cancer, photodynamic therapy (PDT), targeted delivery, titanium peroxide

## Abstract

**Background:**

Although photodynamic therapy (PDT) has emerged as a potential alternative to conventional chemotherapy, the low reactive oxygen species (ROS) yield of the photosensitizer such as TiO_2_ nanoparticles has limited its application. In addition, it is difficult to achieve effective tumor treatment with a single tumor therapy.

**Methods:**

We used TiOx nanocomposite (YSA‐PEG‐TiO_X_) instead of TiO_2_ as a photosensitizer to solve the problem of insufficient ROS generation in PDT. Benefiting from the desired mesoporous structure of TiOx, Cantharidin (CTD), one of the active components of mylabris, is loaded into TiOx for targeted combination of chemotherapy and PDT. The cellular uptake in human non‐small cell lung carcinoma cell line (A549) and human normal breast cell line (MCF 10A) was evaluated by confocal microscopy. in vitro cytotoxicity was evaluated using Cell Counting Kit‐8 assay. The ROS was detected via a chemical probe DCFH‐DA and the photodynamic treatment effect of YSA‐PEG‐TiOx was further evaluated by a living‐dead staining. The cell apoptosis was detected by the flow cytometry.

**Results:**

Our findings showed that the modification of YSA peptide improved the cytotoxicity of YSA‐PEG‐TiO_X_/CTD to EphA2 overexpressing A549 non‐small cell lung cancer (NSCLC) than non‐YSA modified counterparts. In addition, TiOx generated adequate ROS under X‐ray irradiation to further kill cancer cells. Flow analysis results also proved the superiority of this combined treatment.

**Conclusions:**

YSA‐PEG‐TiO_X_ nanoparticles could significantly increase ROS production under X‐ray exposure and provide a new drug delivery nanocarrier for CTD in combination with PDT to achieve effective NSCLC treatment.

## Introduction

Cantharidin (CTD), one of the active components of mylabris, is a traditional kind of Chinese medicine, and has been found to be effective against a variety of malignant tumors.^1^, ^2^, ^3^ Many previous studies have reported that the number of white blood cells in peripheral blood and the immunity function of the body were not affected during tumor therapy with CTD.^4^, ^5^, ^6^ Hence, CTD may be a good alternative as a chemotherapeutic agent in cancer therapy. However, several severe side effects caused by CTD such as vomiting, cystitis, nephritis and so on still exist.

As is well known, nanoparticles have been used as the carriers to achieve efficient drug delivery and reduce toxic side effects over the past decade with the rapid development of nanotechnology.^7^, ^8^, ^9^ Reactive oxygen species (ROS)‐mediated photodynamic therapy (PDT) as a nanoparticle aided treatment strategy with high specificity has recently received considerable attention.^10^, ^11^, ^12^, ^13^ PDT commonly means the induced death of cancer cells caused by ROS which is produced with the photosensitive drugs or photosensitizers in the irradiation of fixed wavelength light.^14^, ^15^, ^16^ However, it is difficult to cure cancer with PDT alone. Therefore, a combination of chemotherapy and PDT is expected to significantly improve the efficiency of cancer treatment.

Commonly, traditional PDT as a light‐triggered local treatment cannot attack unirradiated tumors less than 1–2 cm.^17^ In order to overcome the limitations of traditional PDT, various strategies have been explored in recent years.^18^ Among them, the application of deep tissue penetrating X‐ray photosensitizers has shown great potentials in improving the penetration depth of conventional PDT. Titanium dioxide (TiO_2_), with a stable mesoporous structure, large surface area, and tailorable pore size has proven to be a suitable photosensitizer for PDT.^19^, ^20^ However, it produces limited levels of ROS under X‐ray exposure. Titanium peroxide (TiOx), a product formed after oxidation of TiO_2_, has been recently developed and exhibited a superior ability to generate ROS.^21^

Eph receptors, as the largest tyrosine kinase receptor family, consist of two Eph classes (EphA and EphB). EphA2, a 130‐kDa, 976‐amino acid transmembrane glycoprotein, is highly expressed in many human cancers such as non‐small cell lung cancer (NSCLC) and can be treated as a new target for drug delivery and cancer treatment.^22^, ^23^, ^24^ In our previous work, the YSA peptide with YSAYPDSVPMMSK sequence has been proven to be a targeting motif that mediates drug delivery to tumor cells expressing EphA2.^25^ The structural formula of YSA is shown in Figure S1.

PEG is a widely used modification in the application of nanomaterials, mainly due to its great enhancement on the stability of the nanoplatform and blood circulation time, which increases the tumor accumulation after systemic administration.^26^, ^27^, ^28^

**Figure 1:**
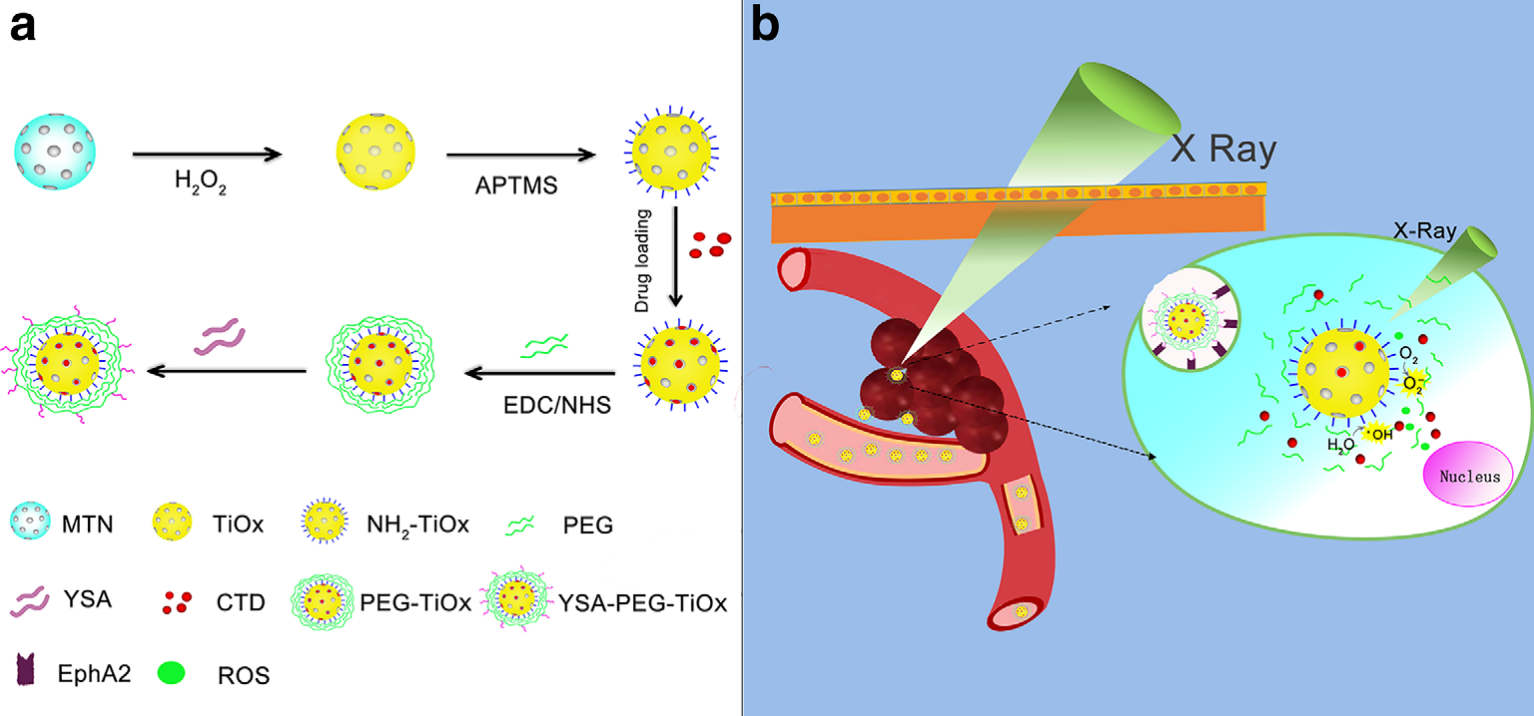
(**a**) Schematic illustration of the preparation of YSA‐PEG‐TiOx/CTD. (**b**) Schematic illustration of YSA‐PEG‐TiOx/CTD for EphA2 targeting drug delivery and the photodynamic effect upon X‐ray irradiation.

Based on the above background, in this study a nanodrug delivery system YSA‐PEG‐TiOx/CTD was developed and studied on A549 human NSCLC cell line. As shown in Figure 1, the TiOx, as a photosensitizer and drug delivery carrier, was used to load with CTD. PEG was coated on the surface of the nanomaterial to prevent drug leakage and conjugate YSA to achieve the targeted drug delivery to EphA2 highly expressed NSCLC. It is believed that YSA‐PEG‐TiOx/CTD achieved the antitumor effects by the following steps. First, the nanoparticles were more prone to accumulate in the tumor tissue due to enhanced permeability and retention effects (EPR).^29^, ^30^ Second, this drug delivery system could then be specifically recognized by A549 cells via the crosstalk between YSA and EphA2. Finally, tumor cells were killed due to encapsulated CTD and ROS produced by TiOx under X‐rays.

## Methods

### Materials

Tetrabutyl titanate were obtained from Aladdin (Shanghai, China). (3‐Aminopropyl) trimethoxysilane (APTMS) was purchased from Macklin (Shanghai, China). Titanium butoxide (GC, 99.0%), hydrogen peroxide (H_2_O_2_), heptanoic acid (AR, 98.0%), ethanol, N‐(3‐dimethylaminopropyl)‐N‐ethylcarbodiimide hydrochloride (EDC), doxorubicin (DOX) and N‐hydroxysuccinimide (NHS) were purchased from Aladdin (Shanghai, China). Cantharidin (CTD) was purchased from Sigma (St. Louis, MO, USA). NHS‐PEG_2000_‐COOH was purchased from Ponsure Biotechnology Co., Ltd (Shanghai, China). The BCA Kit and 4′,6‐Diamidino‐2‐phenylindole (DAPI) were obtained from Solarbio (Beijing, China). Cell Counting Kit‐8 (CCK‐8) and DCFH‐DA were purchased from Beyotime Biotechnology Co., Ltd (Shanghai, China). Fetal bovine serum (FBS), RPMI 1640, DMEM, trypsin‐EDTA and penicillin‐streptomycin were obtained from Zhejiang Tianhang Biotechnology Co. Ltd (Zhejiang, China). The YSA peptide (YSAYPDSVPMMSK) was obtained from GL Biochem Peptide Co., Ltd (Shanghai, China). The calcein‐AM/PI double staining kit was obtained from Yeasen (Shanghai, China). Annexin V‐FITC/PI apoptosis double staining kit was obtained from Beyotime Institute of Biotechnology (Shanghai, China). The abbreviations are summarized in Table 1.

**Table 1:** List of abbreviations

Abbreviations	Full name
PDT	Photodynamic therapy
ROS	Reactive oxygen species
CTD	Cantharidin
TiOx	Titanium peroxide
EPR	Enhanced permeability and retention effects
MTNs	Mesoporous titanium dioxide nanoparticles
TEM	Transmission electron microscopy
BET	Brunauer Emmett Teller
BJH	Barett Joyner Halenda
RBCs	Red blood cells
DOX	Doxorubicin
DLS	Dynamic light scattering
PI	Propidium iodide
NSCLC	Non‐small cell lung cancer
APTMS	(3‐Aminopropyl) trimethoxysilane
FT‐IR	Fourier transform infrared spectra
YSA	Peptide: YSAYPDSVPMMSK sequence
CCK‐8	Cell Counting Kit‐8

### Preparation of NH_2_‐TiO_X_

Mesoporous titanium dioxide nanoparticles (MTNs) were synthesized following the guidelines of the published method.^31^ In brief, 50 μL of heptanoic acid and 20 mL of ethanol were added to the beaker and stirred for 5–10 minutes. Next, 300 μL of tetrabutyl titanate was added to the mixture, and it was continually stirred for 15 minutes. Then, 5 mL of ultrapure water was added and the mixture stirred for another 30 minutes. The obtained reaction liquid was transferred to a reaction vessel and hydrothermally reacted at 120°C for 40 minutes. The resulting product was centrifuged, and washed successively with ethanol and ultrapure water three times.

For the synthesis of TiOx, 20 mg of the MTNs was oxidized with 100 μL of H_2_O_2_. The obtained TiOx was washed with ultrapure water three times. Then, for the synthesis of NH_2_‐TiOx, 2 mg of TiOx and 50 μL of APTMS were dissolved in 25 mL of ethanol. The mixture was sonicated to disperse uniformly. The reaction product was then washed successively with ethanol and ultrapure water three times and dispersed in ultrapure water for preservation.

### Synthesis of YSA‐PEG‐TiOx/CTD

A total of 10 mg of NH_2_‐TiOx and 4 mg of CTD were dissolved in 4 mL of 95% ethanol and sonicated to disperse well. The mixture was then stirred at room temperature for 24 hours in the dark. The nanoparticles were washed with 95% ethanol three times, and analyzed with an ultraviolet‐visible (UV‐vis) spectrometer to verify the successful loading of CTD. Next, 4 mg of NHS‐PEG_2000_‐COOH was hydrated for 24 hours, followed by activation with EDC and NHS for two hours. To obtain the PEG‐TiOx/CTD NPs, 10 mg of NH_2_‐TiOx/CTD was added to the activated PEG solution and stirred for 24 hours. The coupling ratio of PEG was calculated by measuring the unconjugated PEG in the supernatant using the reagent of barium chloride for a spectrophotometric method. The same method was used to get YSA‐PEG‐TiO_X_/CTD. The coupling ratio of YSA was calculated by measuring unconjugated YSA in the supernatant using the BCA Assay Kit. Finally, the nanoparticles were washed with PBS (pH 7.4) three times and stored in PBS for further use.

### In vitro drug release

The in vitro release behavior of CTD from nanoparticles (TiOx/CTD or YSA‐PEG‐TiOx/CTD) was studied using a dialysis method. Briefly, the nanoparticles were suspended in PBS of two different pH values (5.0 and 7.4), respectively: the concentration of nanoparticles was 1 mg/mL. The mixture was then placed in a dialysis bag (M_W_ = 14 000) and the release medium was equilibrated with 60 mL of the corresponding PBS buffer at 37°C under horizontal shaking (100 rpm/minute). Then 50 μL of the solution was withdrawn from the buffer medium and replaced with an equal volume of fresh medium at predetermined time points. The amount of released CTD was measured using a UV‐vis spectrometer at 202 nm.

### Characterization

The particle size, potentials and polydispersity (PDI) of the synthesized TiOx, NH_2_‐TiOx, PEG‐TiOx and YSA‐PEG‐TiOx were measured by dynamic light scattering (DLS) analysis using a Malvern Zetasizer Nano ZS (Malvern, UK). Transmission electron microscopy (TEM) was carried out to observe the morphology of the above nanoparticles using an FEI Tecnai G2 F30 instrument equipped with EDX (Bruker super‐X). Nitrogen adsorption‐desorption measurements were carried out in a liquid nitrogen atmosphere and samples were outgassed for six hours before the measurements were taken. The surface area and pore size distribution of the samples were measured by Brunauer Emmett Teller (BET) and Barett Joyner Halenda (BJH) measurements. FT‐IR spectra were measured using a Fourier transform infrared spectrometer (FT‐IR, Nicolet iN10 MX & iS10, ThermoFish) with KBr pellets. An ultraviolet‐visible (UV‐vis) spectrometer (Thermo Scientific Instrument Co., Ltd) was used to analyze the content of CTD.

### Hemolysis assay

Fresh whole blood was obtained from the ear vein of rabbits. Red blood cells (RBCs) were isolated by centrifugation at 1000 rpm for 20 minutes at 4°C and washed three times with PBS. Then 1 mL of the nanoparticle (TiOx, PEG‐TiOx or YSA‐PEG‐TiOx) suspension in PBS at different concentrations was placed in centrifuge tubes. Ultrapure water was used as the positive control and PBS was used as the negative control. After 0.5 hours of incubation in the water bath at 37°C, 0.1 mL of the RBC suspension was added. After two hours of incubation at 37°C under constant shaking, the suspensions were centrifuged at 1000 rpm for 10 minutes. The color of the supernatant in each centrifuge tube was observed to determine the hemolytic phenomenon, and 200 μL of the supernatant from each tube was added into a 96‐well plate. The absorbance (OD value) of each sample was measured using a microplate reader at a wavelength of 576 nm. The hemolysis percentage was calculated using the following equation:

Hemolysis (%) = (OD576 sample ‐ OD576 negative control)/(OD576 positive control ‐ OD576 negative control) × 100%.

### Cell culture

The human non‐small cell lung carcinoma cell line (A549) and human normal breast cell line (MCF10A) were purchased from the Institute of Basic Medical Science, Chinese Academy of Medical Sciences (Beijing, China). A549 cells were incubated in complete RPMI 1640 (RPMI 1640 with 10% [v/v] FBS, 100 U/mL of penicillin and 100 mg/mL streptomycin) at 37°C in a humidified atmosphere containing 5% CO_2_. MCF10A cells were cultured in complete DMEM medium (DMEM with 10% (v/v) FBS, 100 U/mL penicillin and 100 mg/mL streptomycin) at 37°C in a humidified atmosphere containing 5% CO_2_.

### Cellular uptake assay

To test the cellular internalization of YSA‐PEG‐TiOx/CTD nanoparticles, doxorubicin (DOX) was used to replace CTD due to its autofluorescence properties. The confocal microscopy was used to study the uptake of nanoparticles (PEG‐TiOx/DOX and YSA‐PEG‐TiOx/DOX) by A549 cells and MCF10A cells. In brief, glass slides were placed in 24‐well plates and cells were incubated to grow on the glass coverslips for 24 hours. The cells were then incubated with free DOX, PEG‐TiOx/DOX or YSA‐PEG‐TiOx/DOX (containing 10 μg/mL DOX) for three hours. The cells were subsequently washed three times with cold PBS and fixed with 4% paraformaldehyde. Finally, the cell nuclei were stained with DAPI (10 ng/mL) and imaged under a laser confocal fluorescence microscope (LSCM, Leica, TCS SP8, Germany).

### Detection of intracellular ROS

DCFH‐DA Reactive Oxygen Species Assay Kit was used to detect the intracellular ROS production under X‐ray irradiation. A549 cells were seeded in six‐well plates. After incubation with MTN, TiOx, YSA‐PEG‐TiOx (10 mg/mL) for 24 hours, DCFH‐DA was added and incubated for a further hour. The cells were then washed twice with PBS and exposed to X‐rays for 0.5 hour. X‐ray irradiation was performed using a BJI‐1 (Xianwei, Shanghai, China) at a voltage of 60–75 kV and a current of 0.15–0.35 mA. After another 0.5 hour of incubation, fluorescence images of the treated cells were acquired using an inverted fluorescence microscope (Leica DMI 4000B).

### Evaluation on therapeutic effects of PDT in vitro

A549 cells were seeded in 24‐well plates at a density of 5 × 10^4^ cells per well and incubated overnight. The cells were then incubated with different nanoparticles (MTN, TiOx or YSA‐PEG‐TiOx) for 24 hours. After incubation, the cells were washed three times with PBS and irradiated with X‐rays for 0.5 hour. Finally, the irradiated cells were rinsed with PBS and stained with calcein‐AM (2 mM) and PI (1.5 mM), respectively. After 0.5 hour incubation, the stained cells were observed by inverted fluorescence microscope (Leica DMI4000B).

### In vitro cytotoxicity assay

The toxicity of the materials to cells was investigated using a CCK‐8 assay. Briefly, A549 cells were seeded in 96‐well plates at a density of 5 × 10^3^ cells per well and incubated overnight. A549 cells were incubated with different nanoparticles (MTN, TiOx, PEG‐TiOx or YSA‐PEG‐TiOx) or CTD‐loaded groups (CTD, TiOx/CTD, PEG‐TiOx/CTD or YSA‐PEG‐TiOx/CTD), with or without X‐ray irradiation for 30 minutes. After 24 hours of incubation, 10 μL of CCK‐8 was added to each well and the cells were incubated for one hour. The absorbance was measured by a microplate reader (BioTek, USA) at a wavelength of 450 nm.

### Cell apoptosis assay

A549 cells was seeded in 6‐well plates (5 × 10^4^ cells/well) and incubated overnight. After that, the culture media was discarded and cells were incubated with CTD, PEG‐TiOx/CTD or YSA‐PEG‐TiOx/CTD (containing 2 μg/mL CTD) for 36 hours. For the X‐ray group, A549 cells were exposed to X‐ray irradiation for 30 minutes after 12 hours of incubation. Flow cytometric analysis was then carried out to detect the proportion of apoptotic cells using an Annexin V‐FITC/PI apoptosis double staining kit.

### Statistical analysis

All experiments were repeated at least three times. Data were presented as mean ± SD. Statistical significance was evaluated using two‐tailed heteroscedastic Student's *t*‐test (*, *P* < 0.05; **, *P* < 0.01).

## Results and discussion

### Synthesis and characterization of functionalized TiO_x_

The synthesis of YSA‐PEG‐TiOx/CTD was divided into six steps. (i) Based on previous work, MTN was synthesized by a hydrothermal method. These uniform MTN spheres have diameters in the range of 100–200 nm. (ii) TiOx was then prepared through oxidizing MTN according to the method described in the literature.^21^ (iii) NH_2_‐TiOx was obtained from TiOx modified with APTMS. (iv) CTD was loaded into the NH_2_‐TiOx by diffusion in an aqueous medium under mechanical agitation. (v) The amino group in NH_2_‐TiOx/CTD was conjugated with the carboxyl group in PEG through an amide bond to form PEG‐TiOx/CTD. (vi) The amino group in PEG‐TiOx/CTD was conjugated with the carboxyl group in YSA through an amide bond to form YSA‐PEG‐TiOx/CTD.

**Figure 2:**
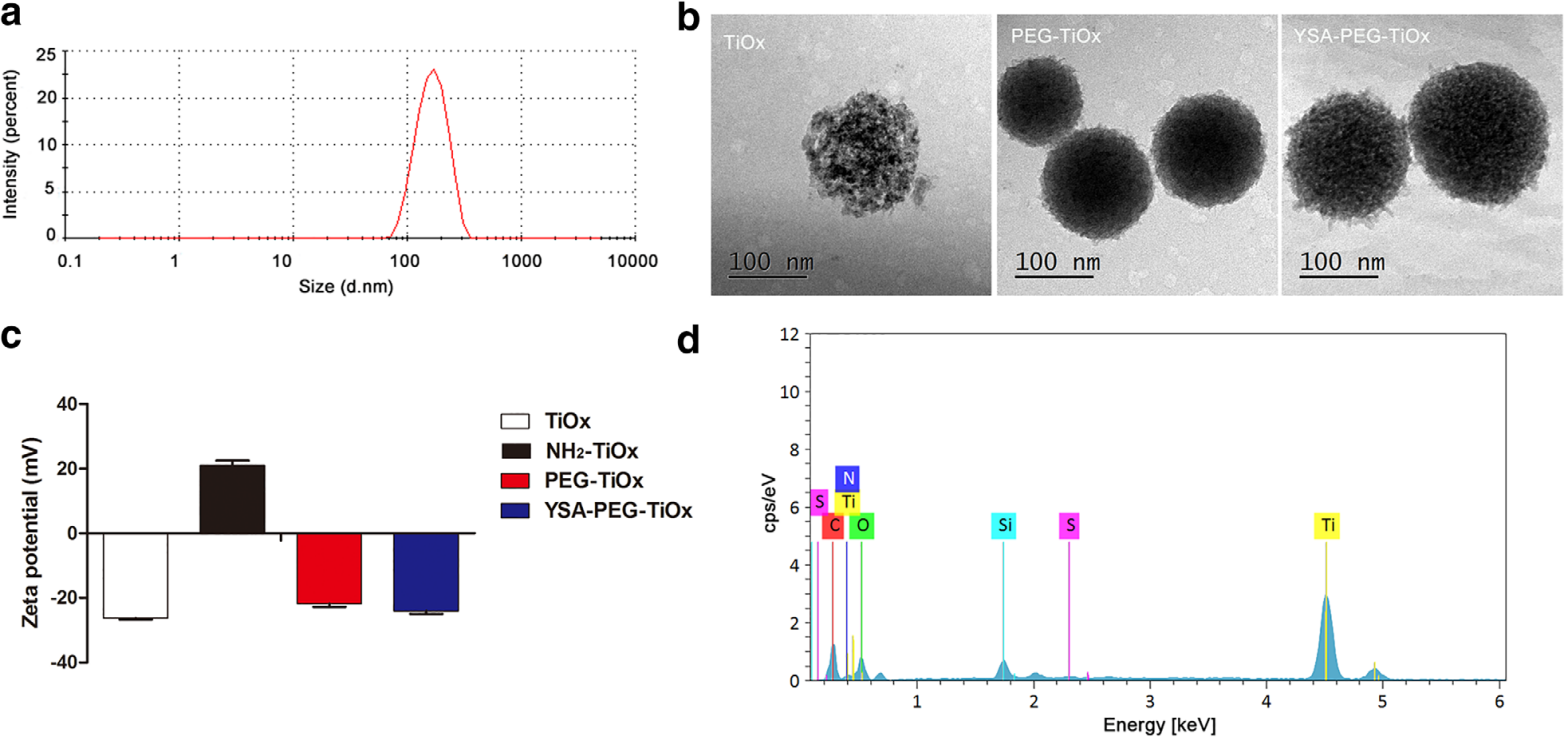
(**a**) Size distribution of YSA‐PEG‐TiOx. (**b**) TEM micrographs of TiOx, PEG‐TiOx and YSA‐PEG‐TiOx. (**c**) Zeta potentials of TiOx, NH_2_‐TiOx, PEG‐TiOx and YSA‐PEG‐TiOx. TiOx 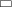, NH_2_‐TiOx 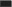, PEG‐TiOx 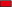, YSA‐PEG‐TiOx 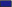 (**d**) Energy spectrum analysis of YSA‐PEG‐TiOx.

**Figure 3:**
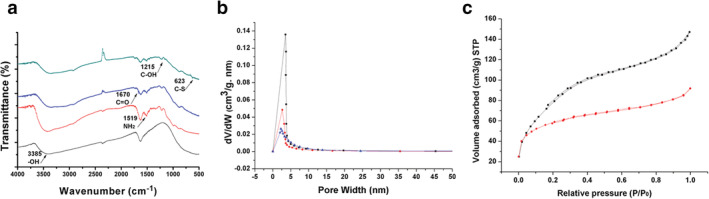
(**a**) FT‐IR spectroscopy of TiO_X_, NH_2_‐TiO_X_ PEG‐TiO_X_ and YSA‐PEG‐TiO_X_. 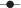
TiOx, 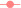
NH_2_‐TiOx, 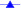
PEG‐TiOx and 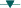
YSA‐PEG‐TiOx (**b**) Pore size distribution of TiOx, PEG‐TiOx and YSA‐PEG‐TiOx from BJH adsorption. 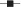
TiOx, 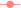
PEG‐TiOx and 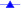
YSA‐PEG‐TiOx (**c**) The nitrogen adsorption/desorption isotherms of TiOx and YSA‐PEG‐TiOx. 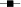
TiOx, 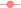
YSA‐PEG‐TiOx

The morphology, particle size, and zeta potential properties of the functionalized MTNs were characterized and are shown in Figure 2a. The particle size of YSA‐PEG‐TiOx was approximately 150 nm in the dynamic light scattering (DLS) measurements. The morphology of both MTN and TiOx was spherical, and the particle size did not change significantly (Figure S2). Also, the TEM images of TiOx, PEG‐TiOx and YSA‐PEG‐TiOx all exhibited a high pore structure (Figure 2b), which provided the possibility for drug loading. The successful construction of YSA‐PEG‐TiOx was validated via zeta potential, energy spectrum and Fourier transform infrared spectra (FT‐IR). As shown in Figure 2c, the zeta potential of NH_2_‐TiOx was 21 mV. After grafting PEG, the zeta potential was reversed from a positive value to a negative one of −21.77 mV due to the introduction of negatively charged carboxyl groups of PEG. This result indicated the successful conjugation of PEG to NH_2_‐TiOx. As shown in Figure 2d, the S element was newly added in the energy spectrum of YSA‐PEG‐TiOx, which proved that YSA was successfully conjugated to the surface of TiOx because the sole source of S element was YSA. Then, the FT‐IR spectroscopy was used to further confirm the structure of YSA‐PEG‐TiOx. As illustrated in Figure 3a, the absorption peaks at 1519 cm are assigned to the NH_2_ stretching of NH_2_‐TiOx. In addition, due to the presence of PEG, the absorption peak of the carboxyl group (C = O) was observed at 1670 cm. For YSA‐PEG‐TiOx, the absorption peaks at 1215 cm and 623 cm (phenolic hydroxyl C‐OH and S‐C stretching vibrations in YSA, respectively) were observed. This result demonstrated the successful construction of YSA‐PEG‐TiOx.

In addition, the pore size of original TiOx was 3.6 nm, while the pore size of PEG‐TiOx and YSA‐PEG‐TiOx decreased to 2.6 nm and 2 nm (Figure 3b), which indicated that TiOx was coated with PEG or YSA. The nitrogen adsorption/desorption isotherms of TiOx and YSA‐PEG‐TiOx are shown in Figure 3c. The two samples showed type IV isotherms, further indicating the mesoporous structure of the nanoparticles. The monolayer adsorption capacities of TiOx and YSA‐PEG‐TiOx were 69.5 mL and 34.2 mL, respectively.

### Drug loading and pH‐responsive drug release

**Figure 4:**
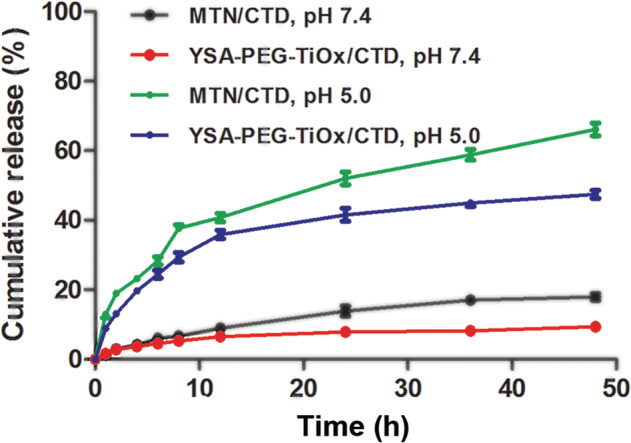
The cumulative release of CTD from TiOx/CTD, YSA‐PEG‐TiOx/CTD at pH 7.4 and pH 5.0. Data are presented as mean ± SD (*n* = 3). 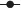 MTN/CTD, pH 7.4, 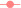 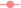 YSA‐PEG‐TiOx/CTD, pH 7.4, 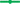 MTN/CTD, pH 5.0, 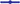 YSA‐PEG‐TiOx/CTD, pH 5.0

Effective encapsulation of drugs and effective release in tumors are essential for nanodrug delivery systems. The CTD loading content and encapsulation efficiency were calculated to be 23.75% and 92.33%, respectively. As shown in Figure 4, the drug release at different pH was studied. In all drug release profiles, there was a positive correlation between drug release and time. In the release medium of pH 5.0, the cumulative release of CTD increases dramatically. The above results indicate that the materials with a certain pH sensitivity will be more conducive to the safety of nanodrug delivery systems and the killing of tumor cells.^32^ Interestingly, the cumulative release of YSA‐PEG‐TiOx/CTD is much lower than that of MTN/CTD, which is mainly due to the extended drug release by PEG modification. In this way, YSA‐PEG‐TiOx/CTD can maintain a small amount of drug release during normal tissue circulation in the body. After reaching the tumor site, it can release a large amount of drug in the slightly acidic environment because of pH response characteristics. In addition, the presence of PEG allows the loaded drug to release for a longer period of time, achieving the purpose of treating tumors.

### Toxicity studies

**Figure 5:**
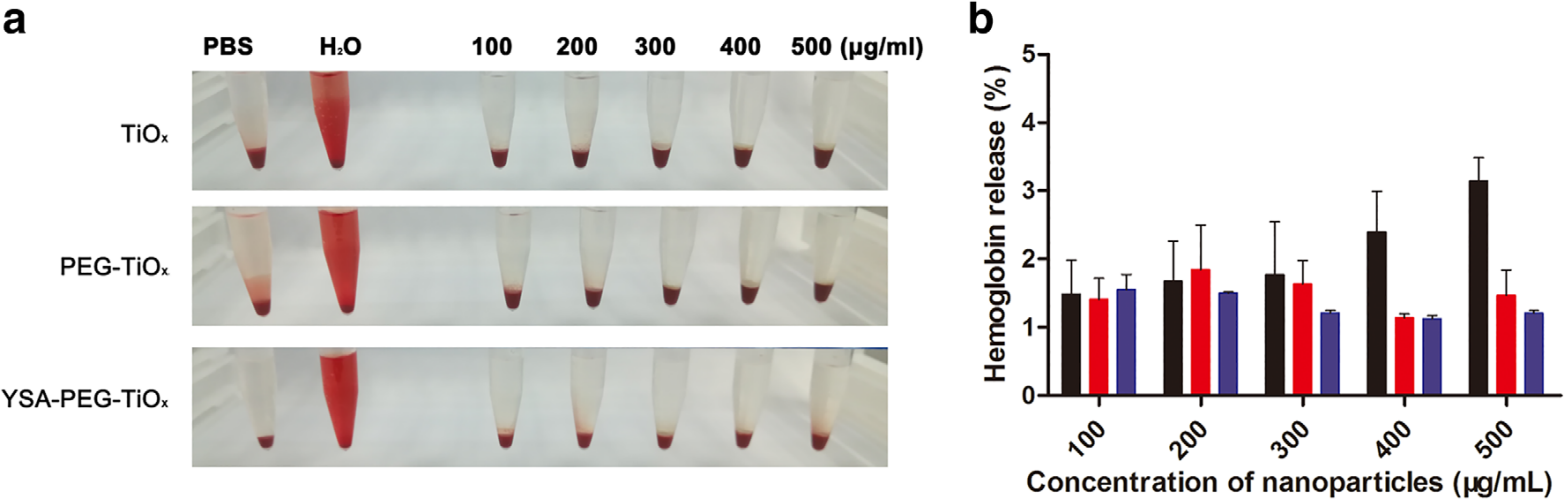
(**a**) Hemolysis study for TiOx, PEG‐TiOx and YSA‐PEG‐TiOx. (**b**) The hemolysis rate in RBCs upon two hours incubation with different nanoparticles at incremental concentrations, data are mean ± SD (*n* = 3). 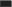 TiOx, 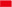 PEG‐TiOx, 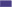 YSA‐PEG‐TiOx

The nanoparticles interact with blood components when they are applied to the blood.^33^ The cell and protein‐related functions in the blood may be altered by the physicochemical properties of the nanoparticles, such as morphology, size, surface area, surface charge and composition.^34^ Therefore, an assessment of the blood contact properties of nanomaterials, such as hemolytic activity, is critical to evaluate the safety of its clinical application. The degree of hemolysis caused by TiOx, PEG‐TiOx and YSA‐PEG‐TiOx exposed to diluted rabbit blood is shown in Figure 5a. There was no obvious hemolysis in the nanoparticle groups. The percentage of hemolysis remained below 4% in the range of concentrations tested (Figure 5b), and a percent hemolysis of less than 5% according to ASTME 2524‐08 indicated that the test materials were nonhemolytic. Good blood compatibility could ensure the safety of YSA‐PEG‐TiOx/CTD for further clinical research.

### In vitro cellular uptake studies

**Figure 6:**
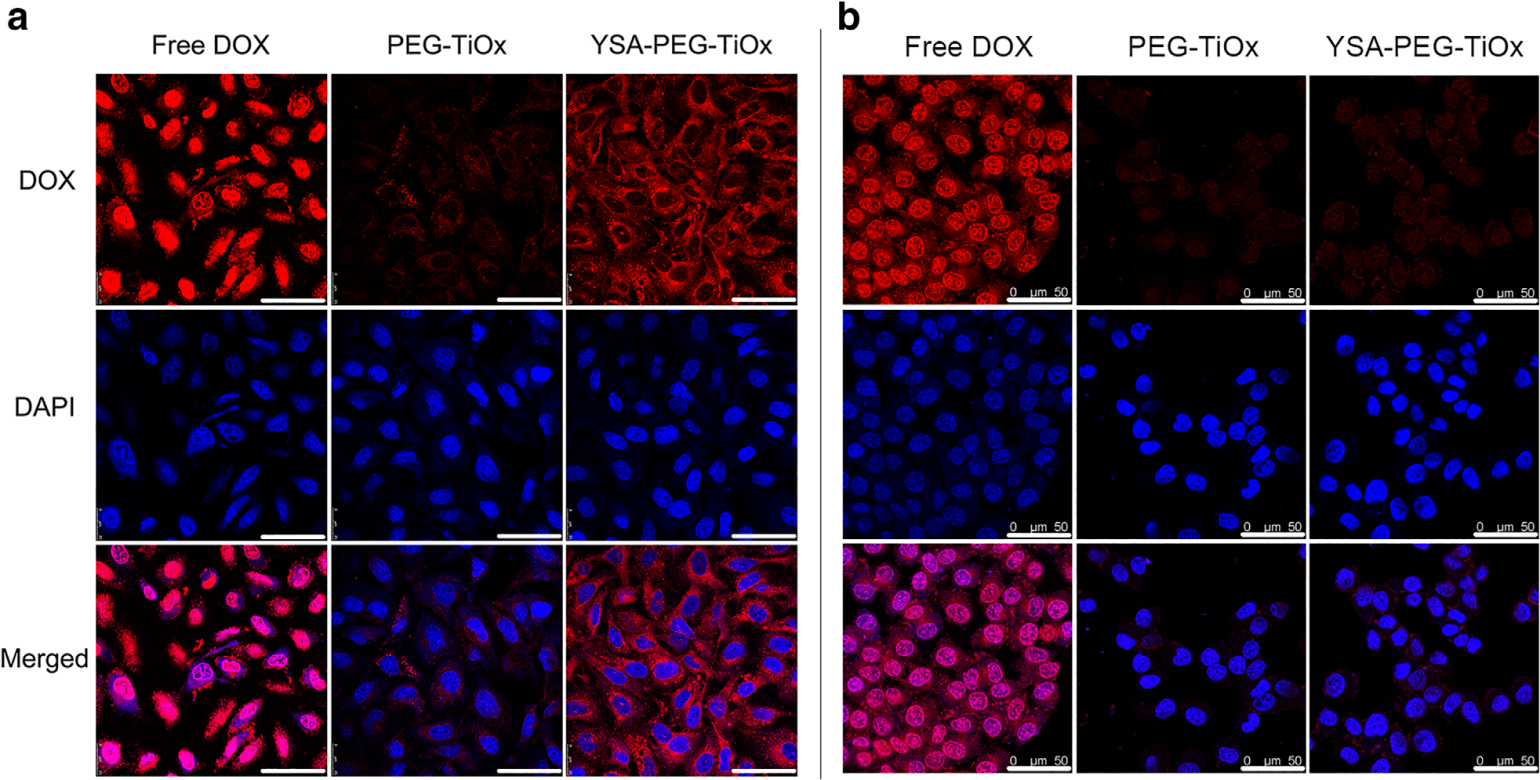
Confocal laser‐scanning microscopy images of A549 cells (**a**) and human normal breast MCF10A cells (**b**) incubated with free DOX, PEG‐TiOx/DOX, YSA‐PEG‐TiOx/DOX at 37°C for three hours. Scale bars represent 50 µm.

Efficient cellular uptake is a major requirement for the therapeutic efficacy of nanoparticles.^35^, ^36^ To investigate whether YSA‐PEG‐TiOx/CTD could enhance the uptake in EphA2‐overexpressing A549 cells, cell uptake studies were performed qualitatively by confocal microscopy. Auto‐fluorescent DOX replaced CTD in the nanoparticles so that the nanoparticles could emit fluorescence for detection. The red fluorescence in Figure 6 indicated the distribution of DOX‐loaded nanoparticles in the cells and blue fluorescence indicated the nuclei stained by DAPI. The results revealed that the cells incubated with free DOX alone showed strong red fluorescence in the nucleus. This is mainly because free DOX could rapidly enter the nucleus to bind DNA.^37^ Furthermore, it can be observed that the fluorescence intensity in A549 cells incubated with YSA‐PEG‐TiOx/DOX was stronger than that in A549 cells incubated with PEG‐TiOx/DOX (Figure 6a), whereas in human normal breast MCF10A cells, there was no significant difference between YSA‐PEG‐TiOx/DOX and PEG‐TiOx/DOX groups (Figure 6b). These results suggested that the modification of YSA on the surface of nanoparticles enhanced the cellular uptake in EphA2‐overexpressing A549 cells.

### Detection of reactive oxygen species (ROS)

**Figure 7:**
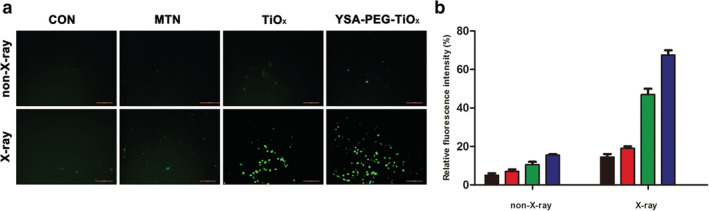
Intracellular ROS generated by different nanoparticles under X‐ray irradiation. (**a**) Confocal laser‐scanning microscopy images of A549 cells after incubation with nanoparticles in the absence or presence of X‐rays by DCFH‐DA staining. Scale bars represent 200 µm. (**b**) The quantitative results of A549 cells upon two hours incubation with nanoparticles in the absence and presence of X‐ray. 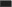 CON, 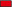 MTN, 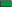 TiOx, 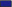 YSA‐PEG‐TiOx.

The ROS was detected via a chemical probe DCFH‐DA to measure the intensity of photoluminescence, which was produced by the fluorescent DCF.^38^ As shown in Figure 7a, the intensity of DCF fluorescence was detected. In the X‐ray group, a significant green fluorescence could be observed in the MTN, TiOx and YSA‐PEG‐TiOx groups. This result indicated that the nanomaterials could produce ROS under X‐ray irradiation. Moreover, the strongest green fluorescence was observed in the YSA‐PEG‐TiOx group, suggesting its highest ROS generation capability and cytotoxicity to cancer cells under X‐ray irradiation. At the same time, it means that cells took up more YSA‐modified nanoparticles, which is in accordance with the improved cellular uptake of YSA‐PEG‐TiOx due to YSA modification. In addition, it is worth noting that the TiOx showed a stronger ROS production capacity than MTN. This demonstrates that improved TiOx has greater potential to kill cancer cells in PDT treatment. In the non‐X‐ray group, no obvious green fluorescence was observed, suggesting that X‐ray is a necessary condition for the production of ROS. The nanoparticles that are not exposed to X‐rays are safe and nontoxic in the systemic circulation. Figure 7b shows the quantitative results. The level of ROS in the cells is significantly increased after X‐ray irradiation.

### Therapeutic effect of PDT of different TiOx in vitro

**Figure 8:**
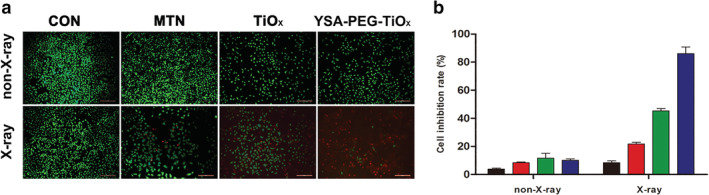
The therapeutic effect of PDT of different nanoparticles in vitro. (**a**) Fluorescence images of A549 cells treated with complete RPMI, MTN, TiOx and YSA‐PEG‐TiOx with or without X‐ray irradiation. Green: Calcein‐AM, live cells; Red: PI, dead cells. Scale bars represent 200 µm. (**b**) The cell inhibition rate was calculated by counting the live/dead cells using imageJ software (*n* = 3). 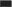 CON, 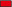 MTN, 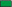 TiOx, 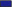 YSA‐PEG‐TiOx

The photodynamic treatment effect of YSA‐PEG‐TiOx was further evaluated by a living‐dead staining. Live cells and dead cells were stained with Calcein‐AM (green fluorescence) and propidium iodide (PI) (red fluorescence). As shown in Figure 8a, there was a clear, high‐intensity green fluorescence but no red fluorescence observed in the four groups without X‐ray irradiation, indicating that cell viability was not affected. However, in the presence of X‐irradiation, the green fluorescence in the nanomaterial‐containing groups were significantly attenuated and the red fluorescence was enhanced, indicating that MTN, TiOx and YSA‐PEG‐TiOx could generate ROS to induce cell death under X‐ray irradiation. Specifically, it could be observed that the cell viability in YSA‐PEG‐TiOx treated group was significantly lower than other groups, which proved that YSA‐PEG‐TiOx treated group produced the most ROS. The quantitative results (Figure 8b) were in accordance with the results above, further demonstrating the photodynamic effect of YSA‐PEG‐TiOx under X‐ray irradiation.

### In vitro cytotoxicity

**Figure 9:**
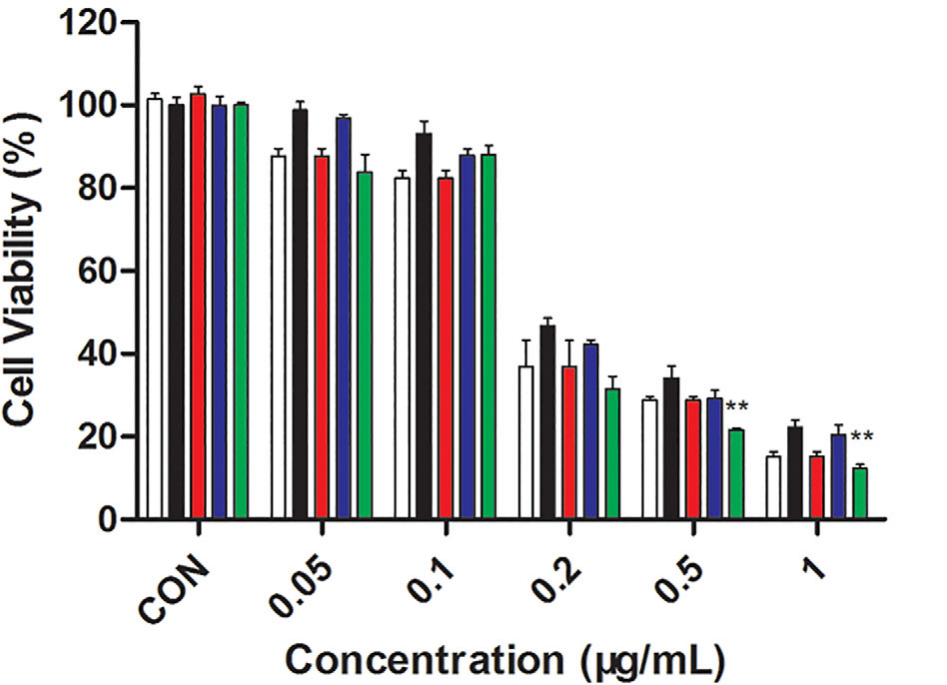
The in vitro cytotoxicity of free CTD, PEG‐TiOx/CTD and YSA‐PEG‐TiOx/CTD with or without X‐ray irradiation (*n* = 4). **, *P* < 0.01. 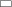 CTD, 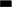 PEG‐TiOx/CTD, 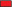 YSA‐PEG‐TiOx/CTD, 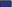 PEG‐TiOx/CTD+X‐ray, 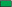 YSA‐PEG‐TiOx/CTD+X‐ray

The effectiveness of therapy was further evaluated using a Cell Counting Kit‐8 assay. First, MTN, TiOx, PEG‐TiOx and YSA‐PEG‐TiOx were tested to verify the safety of the materials. As shown in Figure S3, a high cell viability in different concentrations certified the good biocompatibility of the nanomaterials. The selection of irradiation time has been reported in previous studies.^31^ It can be seen in Figure S4 that TiOx was more toxic to tumor cells than MTN under X‐ray irradiation, which proved that TiOx possessed a better photodynamic effect than MTN. As shown in Figure 9, YSA‐PEG‐TiOx exhibited comparable levels of phototoxicities. YSA‐PEG‐TiOx/CTD under X‐ray irradiation decreased the cell viability substantially to 12.4 ± 1.1%, displaying a remarkable therapeutic efficacy. The above results could be explained due to the following two factors. First, the YSA‐PEG‐TiOx/CTD with enhanced cellular uptake has shown effective PDT effect on A549 cells. Second, the release of antitumor drug CTD in tumor cells further enhances the killing effect. Therefore, YSA‐PEG‐TiOx/CTD with dual advantages of PDT and chemotherapy provides a promising strategy for cancer treatment.

**Figure 10:**
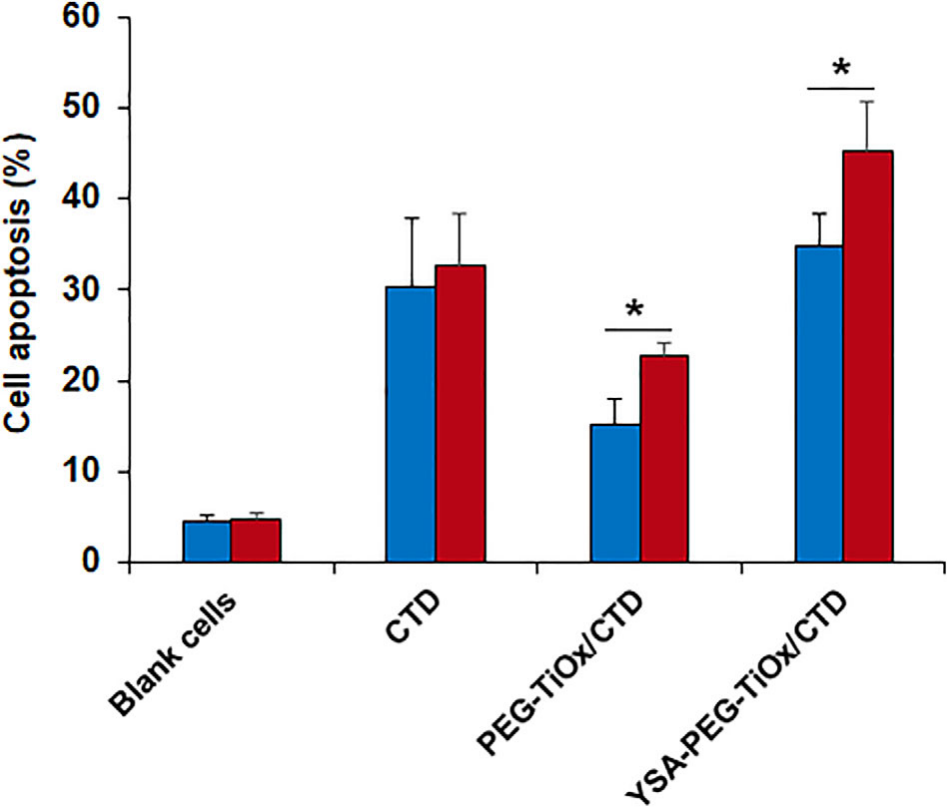
Cell apoptosis detected by flow cytometry of A549 cells incubated with different groups (*n* = 3). *, *P* < 0.05. 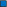 non‐X‐ray, 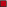 X‐ray.

Meanwhile, the cell apoptosis was detected by flow cytometry. As shown in Figure S5, the viability of cells treated with either MTN or TiOx under X‐ray irradiation decreased. It is notable that the toxicity of the material was significantly enhanced when the antitumor drug CTD was loaded to TiOx (Figure 10). Importantly, YSA‐PEG‐TiOx/CTD caused apoptosis in about 40% of cells under X‐ray irradiation. This may be due to the fact that the ROS produced by TiOx after X‐ray irradiation further increases the killing ability of YSA‐PEG‐TiOx/CTD to tumor cells. The above results proved the superiority of the combination therapy of PDT and chemotherapy. As a new type of treatment system, it is meaningful to conduct further research on YSA‐PEG‐TiOx/CTD.

In conclusion, a new nanoplatform of YSA‐PEG‐TiOx/CTD with chemotherapy and PDT was successfully designed and synthesized in this study. The detected data has shown that TiOx owned outstanding capabilities as a drug carrier and could produce large amounts of ROS when X‐ray irradiated, which was a significant improvement over previous work. The surface coated with PEG prevented the leakage of the model drug CTD. According to cell uptake studies, the targeting peptide YSA on TiOx could achieve a specific recognition of NSCLC cells with high expression of EphA2, resulting in higher cellular uptake of YSA‐PEG‐TiOx in A549 cells. The cytotoxicity experiments and flow cytometry analysis experiments also showed that YSA‐PEG‐TiOx/CTD had stronger cytotoxicity under X‐ray irradiation, suggesting the strong potentiality of antitumor combination of chemotherapy and PDT. This strategy of utilizing our newly designed active targeted drug delivery system may address several limitations of conventional PDT and could be expected to achieve effective NSCLC treatment.

## Disclosure

No authors report any conflict of interest.

## Supporting information


**Figure S1** The structural formula of YSA.
**Figure S2** SEM micrographs of MTN and TiOx.
**Figure S3** The in vitro cytotoxicity of MTN, TiOx, PEG‐TiOx and YSA‐PEG‐TiOx.
**Figure S4** The in vitro cytotoxicity of MTN and TiOx with different concentrations with X‐ray irradiation (*n* = 4).
**Figure S5** Cell apoptosis detected by flow cytometry of (**a**) Blank cells; (**b**) Blank cells+X‐ray; (**c**) MTN; and (**d**) TiO_X_ with X‐ray irradiation.
