# Supplementary material for: Cantharidin‐loaded functional mesoporous titanium peroxide nanoparticles for non‐small cell lung cancer targeted chemotherapy combined with high effective photodynamic therapy
Source: Thorac Cancer. 2020 Apr 4;11(6):1476–86. doi: 10.1111/1759-7714.13414 (PMC7262929; doi:10.1111/1759-7714.13414)
Supplement: Supplementary file 1 — Figure S1 The structural formula of YSA. Figure S2 SEM micrographs of MTN and TiOx. Figure S3 The in vitro cytotoxicity of MTN, TiOx, PEG‐TiOx and YSA‐PEG‐TiOx. Figure S4 The in vitro cytotoxicity of MTN and TiOx with different concentrations with X‐ray irradiation (n = 4). Figure S5 Cell apoptosis detected by flow cytometry of (a) Blank cells; (b) Blank cells+X‐ray; (c) MTN; and (d) TiOX with X‐ray irradiation. [file TCA-11-1476-s001.docx]

**Cantharidin loaded functional mesoporous titanium peroxide nanoparticles for non-small cell lung cancer targeted chemotherapy combined with high effective photodynamic therapy**

Kun Zheng ^a,#^, Runze Chen ^a,#^, Yanxue Sun ^b^, Zhenquan Tan ^c^, Ye Liu ^a^, Xiao Cheng ^a^, Junke Leng ^a^, Zhaoming Guo *^,a^, Pengcheng Xu *^,b^

^a^ School of Life Science and Medicine, Dalian University of Technology, Panjin, Liaoning 124221, China

^b^ Department of Pharmaceutical Engineering, College of Pharmacy, Inner Mongolia Medical University, Hohhot 010110, China

^c^ School of Petroleum and Chemical Engineering, Dalian University of Technology, Panjin, Liaoning 124221, China

* **Corresponding author:**

Department of Pharmaceutical Engineering, College of Pharmacy, Inner Mongolia Medical University, Hohhot 010110, China

Tel: +86-0471-6653132. Fax: +86-0471-6653132. E-mail address: xpc_impu@163.com (Pengcheng Xu)

School of Life Science and Medicine, Dalian University of Technology, Panjin, Liaoning 124221, China.

Tel: +86-427-2631427. Fax: +86-427-2631889. E-mail address: [guozm@dlut.edu.cn](mailto:guozm@dlut.edu.cn) (Zhaoming Guo)

**Author Contributions**

^#^ These authors contributed equally to this work.





**Fig. S1. The structural formula of YSA.**


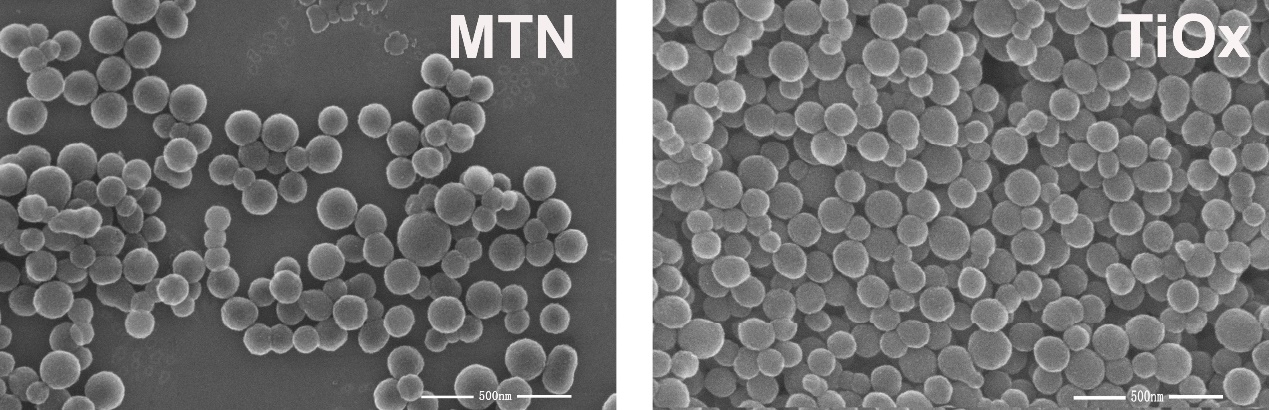


**Fig. S2. SEM micrographs of MTN and TiOx.**


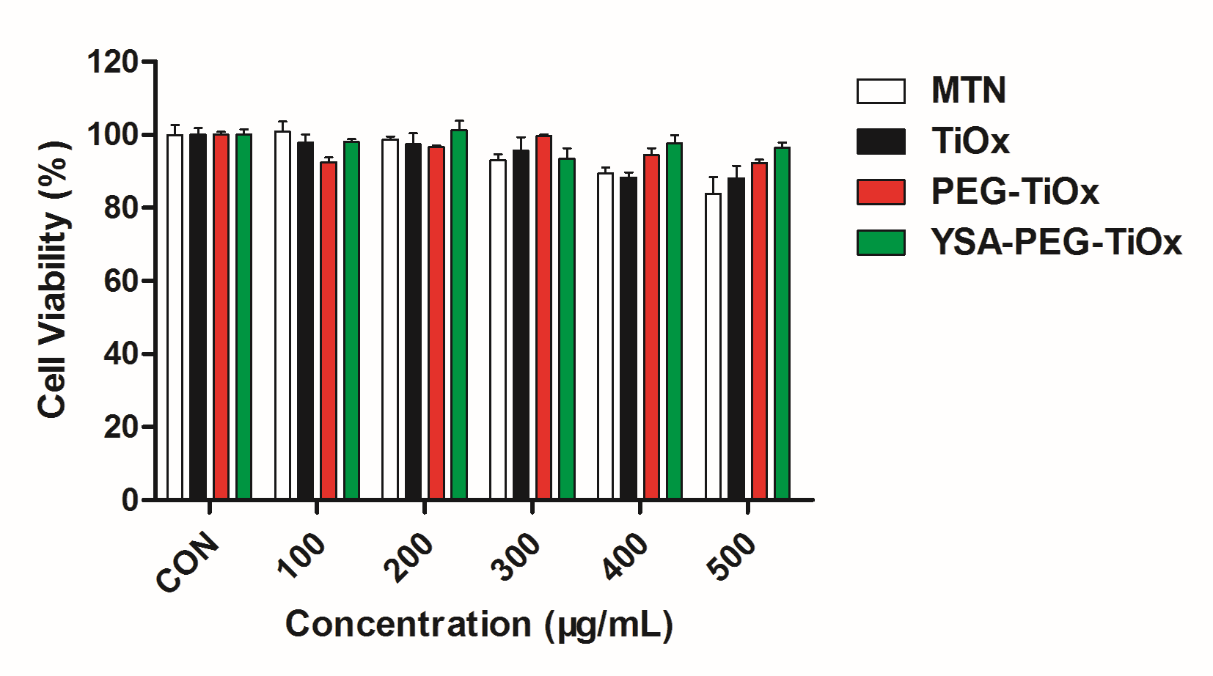


**Fig. S3. The in vitro cytotoxicity of MTN, TiOx, PEG-TiOx and YSA-PEG-TiOx.**


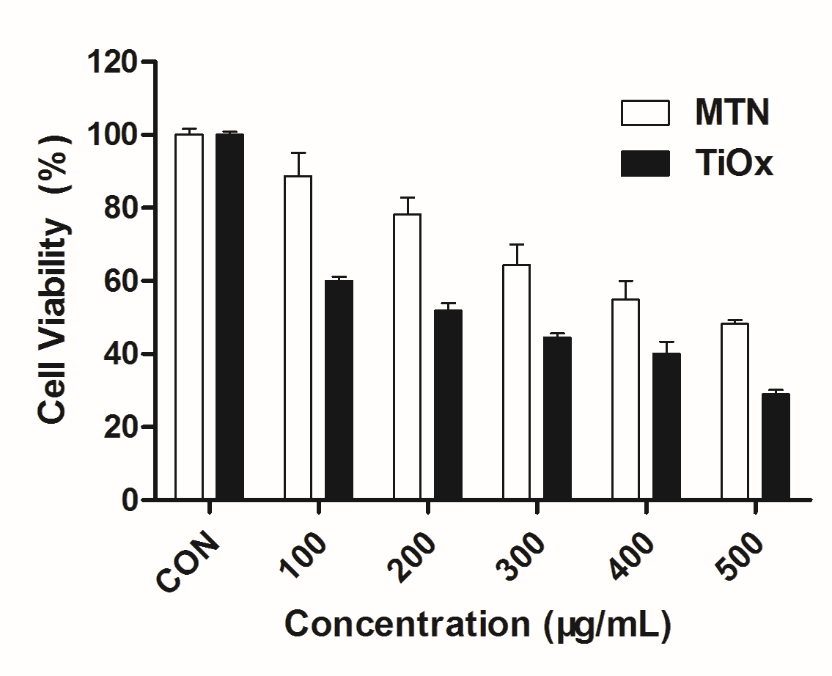


**Fig. S4. The in vitro cytotoxicity of MTN and TiOx with different concentrations with X-ray irradiation (n = 4).**


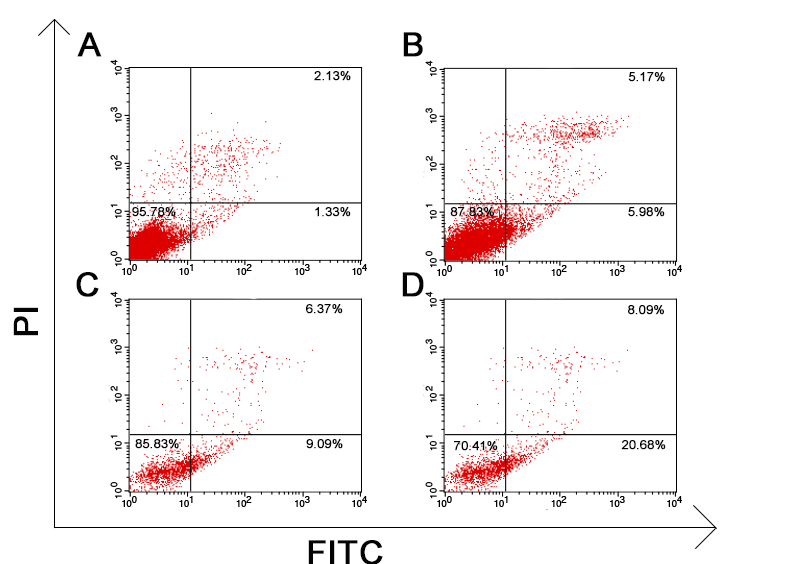


**Fig. S5. Cell apoptosis detected by flow cytometry of A) Blank cells, B) Blank cells+X-ray, C) MTN and D) TiO_X_ with X-ray irradiation.**
